# Modelling the human immunodeficiency virus (HIV) epidemic: A review of the substance and role of models in South Africa

**DOI:** 10.4102/sajhivmed.v19i1.756

**Published:** 2018-02-21

**Authors:** Nathan Geffen, Alex Welte

**Affiliations:** 1 Department of Computer Science, Centre for Social Science Research, University of Cape Town, South Africa; 2 South African Centre for Epidemiological Modelling and Analysis (SACEMA), University of Stellenbosch, South Africa

## Abstract

We review key mathematical models of the South African human immunodeficiency virus (HIV) epidemic from the early 1990s onwards. In our descriptions, we sometimes differentiate between the concepts of a model world and its mathematical or computational implementation. The model world is the conceptual realm in which we explicitly declare the rules – usually some simplification of ‘real world’ processes as we understand them. Computing details of informative scenarios in these model worlds is a task requiring specialist knowledge, but all other aspects of the modelling process, from describing the model world to identifying the scenarios and interpreting model outputs, should be understandable to anyone with an interest in the epidemic.

## Introduction

No epidemic has received the attention of the ongoing human immunodeficiency virus (HIV) and acquired immune deficiency syndrome (AIDS) pandemic, and no matter of public health concern has been the subject of so much controversy and policy debate. Scenario modelling has been widely employed in attempts to better understand the demographic, health and economic impacts of the epidemic under various interventions, for example, antiretroviral treatment, pre-exposure prophylaxis and condom use.

Despite modelling being ubiquitous and some models generating intense public debate, with consequences, for example, on World Health Organization (WHO) treatment and prevention guidelines, it remains poorly understood by non-specialists. Even modellers themselves hold differing views about the principal uses and limitations of models.

This article reviews the evolution of models, and their applications, in the context of the South African HIV epidemic. We describe, in terms aimed at a wider audience than just modellers, the basic structure of the modelling process, challenges that modellers face and how this has affected policy debate. In Appendix 1, we explore in more detail the social complexities of the particular issues and controversies.

## Basic modelling concepts

Books, tutorials and reviews of epidemiological modelling are plentiful, including guidance for working with models in the context of policy debate.^1,2,3,4^ Nevertheless, it is useful to review some essential aspects of all scenario modelling. The aim of mathematical modelling is to first identify the key rules that govern the behaviour of a natural world phenomenon, and then to implement those rules in mathematical relations, so that we can learn more about the phenomenon.

For models of the HIV epidemic, this may mean understanding how gender, age, location and other sociological factors influence fertility, exposure to ‘infectious contacts’, access to healthcare and mortality. What determines whether and what kind of model is feasible or useful are the questions we want to answer about the ‘real world’ epidemic, coupled with the data available to justify assumptions about precisely stated rules driving critical processes (dynamical rules). Mathematical and computational challenges may be substantial and sometimes curtail the ambitions of modellers.

It is important to differentiate the specialised technical aspects of model construction and analysis, carried out by mathematical modellers, from the conceptual aspects, which are accessible to anyone with basic insights into the situation being modelled, including doctors, politicians, health system administrators, biologists and activists. This conceptual and technical distinction helps clarify thinking and reminds us that model building should be an inclusive multidisciplinary process rather than the protected domain of specialists.

For example, in the early 2000s, members of the activist organisation the Treatment Action Campaign approached the developers of the Actuarial Society of South Africa (ASSA) models and asked them to incorporate antiretroviral treatment into their model, which they did. An analysis of the cost of rolling out antiretroviral treatment in the public health system, based on the outputs of the ASSA model, was featured on the front page of the *Mail & Guardian*. The ASSA models were explained by demographers in affidavits in litigation by activists advocating for treatment. The scenarios, assumptions and outputs of the model were debated and understood by a broad range of people: politicians, activists, lawyers, etc. The actual equations in the model spreadsheet were likely of interest to, and understood by, only a handful of specialists.^5,6,7,8^

To maintain the distinction between concepts and techniques, we use concepts popularised by ecological modeller Tony Starfield: *model world* versus *model implementation*.^9^ A Model World is the conceptual realm in which we explicitly declare the rules – usually some simplification of ‘real world’ processes as we understand them. Model Implementation then refers to the mathematical and computational details.

For example, a model world may be conceptually inhabited by genderless people between the ages of 15 and 49 who all have exactly the same behaviours and mortality. We may declare that in our model world each day brings the same risk of infection or death as the day before, without any notion of individual age, the mechanisms of infection or death. A related *model* implementation of such a world may consist of some mathematical equations or computer programme.

Model worlds capture the essential ideas which we then formally analyse and explore in technical investigations, using mathematical and computational tools. A model world has abstracted entities and rules, but no particular history. When we set up initial conditions in a model world, like winding up a clock set to midnight, and then let it run, we produce scenarios – particular realisations of processes and events consistent with the assumptions of the model world. A full-fledged investigation may involve many scenarios located in several model worlds.

There are typically two kinds of variables in a model: (1) *state variables*, that is, scenario-specific accounting indicators, such as the size of population, number of infections and number of deaths, and (2) *parameters*, that is, model world defining metrics such as, most critically, rates of infection, rates of death and other state transition rules. When the model executes (e.g. as a stand-alone piece of software or as a spreadsheet), state variables evolve over time from given initial conditions, but for this to happen, parameter values must actively be chosen. Sometimes parameters are chosen based on pre-existing knowledge or estimates. Sometimes they are chosen entirely heuristically, just to see what is implied by their values lying here or there within some plausible range. Another option is *model calibration*, by which parameters are chosen in such a way that the emergent behaviour of the model is consistent with some data. For example, we can try different values of an infectious ‘contact rate’ (how frequently people become infected), and then see whether a suitably narrow range of this parameter produces a time-varying prevalence that is consistent with survey data.

For sexually transmitted infections, a key aspect of model worlds is how infections occur. Infection can happen for a population group at some rate, without any concern for sexual interactions. There can be a single rate across the population or it could be differentiated by age, risk group and gender. Alternately, infection can be conceptualised at a very fine level of detail: a model world could track sexual relationships – or even sexual acts – per individual, with each individual having their own risk of contracting or transmitting the infection.

In model worlds, there are no grey areas of the kind we find in the real world, no hidden unknown rules, factors and entities – although the interplay of components may be complex and may require some sophistication to implement, or conceptually untangle. Modelling then might be seen as teasing out the implications of hypothetical claims about how the world is composed and governed. If done skilfully, this helps explain some aspect of the real world. It informs real world choices that need to be made, even if the full underlying truth in the real world is much more elusive and ambiguous than in any model world we may have constructed. Table 1 highlights key features of the model worlds implemented in the models we review here.

**TABLE 1:** Examples of models of the South African HIV epidemic.

Model	Model world				Scenarios	Implementation
	Population	Transmission	Mortality	Interventions		
Padayachee and Schall 1990	Black people aged 15 to 49	HIV incidence and prevalence estimated from blood transfusion, antenatal and clinic infection numbers.	Not applicable	None	Used data sources to estimate number of black people aged 15 to 49 with HIV from 1989 to 1991	Three simple models using straightforward calculations
Doyle 1990	Population divided by sex, 5-year age intervals and four HIV risk groups.	Mainly a function of risk group and the proportion of infected people, but ‘some allowance’ for ‘sexual activity according to age and sex’.	Age-related non-HIV mortality. Additional risk of mortality for people with HIV	None	Many. Doyle used it to estimate South African population, while Lee et al. used it to estimate infections in Soweto. The initial HIV-positive population is ‘imported’ into the model.	Macro
Padayachee 1992	Individuals have age and sex.	Each person, adjusted for age and sex, has a probable number of sexual partners with whom they have sex a probable number times, each of whom has HIV with a specified probability.	Mortality not explicitly discussed, but number of AIDS cases calculated based on infection period	None	From 1985 a prespecified number of immigrants with HIV ‘seed’ the model. Number of HIV and AIDS cases estimated until 2000	Micro
ASSA (various)	Population divided by sex, province, 5-year age intervals and four HIV risk groups. Infants enter the population annually. People with HIV at various clinical stages of progression.	Function of risk group, proportion of infected people, age and sex. Mother-to-child transmission also modelled.	Age and sex-related non-HIV mortality. Additional risk of mortality for people with HIV	From ASSA2002, antiretrovirals, mother-to-child transmission prevention, condoms, etc.	Calibrated to available data sources up to the year of the model suffix, and then projected forward.	Macro (originally as spreadsheets, then as C++ code)
Granich deterministic 2009	People of no sex or specific age, except that they are 15 to 49 years. People with HIV are assigned to a WHO stage.	Homogenous: no risk groups, single incidence rate for the whole population.	Single mortality rate for people without HIV. Additional risk of mortality for people with HIV	Scaled-up universal test-and-treat versus treating at CD4 count of 350 versus no treatment	Calibrated to South African adult HIV epidemic.	Macro (the authors also did a stochastic model)
Hontelez 2015	In the most complex model of their nine models, people are differentiated by age and sex.	Heterogeneous sexual behaviour. People are part of sexual networks and people at different stages of HIV infection have different degrees of infectiousness.	Age and sex-related non-HIV mortality. Additional risk of mortality for people with HIV	Similar to Granich et al.	Calibrated to South African adult HIV epidemic.	Micro
THEMBISA 2014–2016	Population divided by sex, province, 5-year age intervals and HIV risk groups. Infants enter the population annually. People with HIV at various CD4 count based stages of progression.	Function of risk group, proportion of infected people, age and sex. Mother-to-child transmission also modelled.	Age and sex-related non-HIV mortality. Additional risk of mortality for people with HIV	Antiretrovirals, PMTCT Option B+, condoms, etc.	Calibrated similarly to ASSA but also includes additional data on marriages and partnerships.	Macro

The crucial point is as follows: everyone with a legitimate interest in the situation being modelled is entitled to a comprehensible description of the model world. They should expect to be part of the model world construction, critique and interpretation processes. Modellers need to talk in conceptual terms about this model world, without resorting to jargon or specialised techniques.

## The core demographic models

Padayachee and Schall, working for Johannesburg’s City Health Department, published the first serious model of the whole South African HIV epidemic in April 1990.^10^ They cited two earlier models that estimated the number of gay men and antenatal care attendees in what was then southern Transvaal with HIV. They also mentioned a WHO model that estimated the number of AIDS cases in South Africa but noted that the model was based on ‘very little, if any, supporting evidence from South Africa’ (p. 330).

Padayachee and Schall actually implemented three simple models, which used antenatal clinic, blood transfusion, sexually transmitted infection and family planning clinic data and population estimates by province to estimate infections for the whole country up to 1992. Their model worlds consist of adult (aged 15 to 49 years) black people, possibly living in a particular province or urban or rural area, but with no other identifiable characteristics. Their first model fitted clinic and blood transfusion data to estimate a rate at which the epidemic was growing. They extrapolated this to calculate national prevalence and the rate at which it was growing up to 1992. Their second model, whose method they called ‘direct’, used various data sets to estimate the number of people with HIV in each province, which they then aggregated for the whole country. Their third model, whose method they called back calculation, used the number of known AIDS cases and an assumption about the time from HIV infection to AIDS to back-calculate the number of HIV cases, derive an incidence rate and then use this to project the number of HIV cases in the future.

They estimated the number of black South Africans, aged 15 to 49 years, with HIV for the end of 1989, 1990 and 1991. Their model calculated between 45 000 and 63 000 infections by end of 1989, rising to between 317 000 and 446 000 at the end of 1991. Clearly there were problems with their methodology: for one thing blood donor HIV prevalence rates were not representative. However, their models provided some idea of the extent of the epidemic using the limited data available then. They wrote: ‘Because of the lack of basic data, these forecasts are tentative, but they nevertheless indicate the great seriousness of the HIV epidemic in South Africa’ (p. 329).

In October 1990, Doyle and Millar, working for the Metropolitan Life Insurance Company, published one of the most influential models of the epidemic.^11^ They constructed a model world with an adult population comprising four risk groups: (1) people having no sexual contact, or in long-term monogamous relationships, who are not at risk of HIV, (2) people at some risk, conceived as being in stable relationships but with one or the other partner having more than one sexual relationship, (3) people with higher levels of risk, such as those with other sexually transmitted infections, and (4) sex workers and people with large numbers of sexual partners. These four risk groups remained a part of highly cited models derived from or based on the Doyle model (as it came to be known) until the late 2000s.

Model world inhabitants were assigned rates for forming new relationships, within and across risk groups, and rates of transmission within relationships. It allowed for 5-year age groups to be defined, with different levels of HIV prevalence at the beginning of a scenario. It also had parameters for fertility, mother-to-child transmission rate and HIV and non-HIV mortality rates. It could be used for heterosexual or homosexual populations, adapted as needed to populations of interest. Doyle applied the model to South Africa, leading to prescient predictions that were not obvious in the early 1990s, for example, that the epidemic would kill many young adults, but that the population would not decline (although the growth rate would slow).^12^ Lee et al. applied the model to Soweto, estimating that by 2010 it would account for 28% – 52% of all deaths there.^13^

The model implementation of Doyle and Millar’s model world was in the form of population counts at discrete time steps, deterministically updated according to the expected values emerging from statistical rules (like probability of infection or death). Doyle cites other models developed by the Institute of Actuaries and Society of Actuaries at the time but points out that these ‘considered one small homogeneous risk group’ and were inappropriate for modelling the South African epidemic.^12^

These model implementations are often called *deterministic compartmental, frequency-dependent* or *macro.* By contrast, a *microsimulation, network* or *agent-based* implementation, possibly of the same underlying model world, proceeds by explicitly tracking a large number of identifiable individual model world inhabitants and subjecting them, usually stochastically, to the different events to which they are exposed. The first microsimulation of the South African epidemic that we can find is a Medical Research Council lecture cited in Doyle and Millar’s 1990 paper. Unfortunately, we can find no further references to this particular one in the literature.

Doyle’s model was a proprietary one used by Metropolitan Life, primarily for the purpose of making decisions about employee benefits (pers comm. Stephen Kramer). It was the progenitor of other models, including those of the ASSA. Given the computing power at the time, the level of detail is impressive, in most respects exceeding the complexity of a widely cited and highly impactful model, published as late as 2009^14^ that stimulated the debate on early treatment as a means of reducing new transmissions.

Groeneveld and Padayachee used a microsimulation implementation of a model world in which each person, based on their age and gender, has an expected number of sexual partners per year, with a specified proportion of ‘short’ relationships, and an estimate of the frequency of sexual contacts with partners who are infected with HIV with a probability dependent on age and gender. The authors also estimated the annual number of immigrants with HIV who entered South Africa annually. Their goal was to ‘to estimate the extent of HIV infection among black heterosexual South Africans’. They attempted to predict new HIV infections for the period 1985–2000 and concluded that there would be 5.7 million people with HIV in South Africa by 2000. By comparison, the most comprehensive up-to-date current model of the epidemic, THEMBISA, estimates that there were 3.3 million people infected in 2000.^15^

Brophy adapted a World Bank model for the South African epidemic and investigated demographic effects on the black population under various scenarios.^16^ The model world divided the population by sex and 5-year age groups. There were also partially overlapping groups: blood transfusion recipients, heterosexual females, heterosexual males and bisexual males. It considered fertility rates, the age pattern of fertility and mortality levels by male and female. The model population was matched to the sex and age structure of the 1985 census. Various data sources were used to estimate fertility and life expectancy. Some parameters, such as the number of sexual partners, coital frequency, condom use, as well as fertility and life expectancy from 2005 to 2010, were essentially guessed (and various scenarios were tried). They calibrated the model so that it estimated the middle estimate of the number of infections in 1990 of the model by Padayachee and Schall (described above). Brophy predicted substantial reductions in the population and life expectancy in 2000, 2005 and 2010 under three AIDS scenarios of increasing severity versus a no-AIDS scenario. In the bleakest scenario, the model estimated that there would be just about 1.9 million people with HIV in the adult black population in 2000 (the actual number was about 3 million).

Dorrington described the origins of the ASSA models.^17^ The first ASSA model was developed on a spreadsheet by a team led by Alan Whitelock-Jones. It was titled ASSA500 and was similar to the Doyle model with some simplifications. Dorrington explains that the motivation for ASSA to develop a model when the Doyle model already existed was that the latter was proprietary and there was a need for a ‘program which the user could alter to his or her needs’ (p. 99). Consequently, the model was placed on ASSA’s website and Dorrington wrote: ‘the reader is encouraged to download and play with it’ (p. 101).

Dorrington also wished to improve the model world of Doyle, specifically because it assumed constant fertility and non-HIV mortality over time; the ASSA model world would include decreasing fertility rates and improving non-HIV mortality. Using the same risk groups as the Doyle model, it additionally accounted for ‘net national in-migration’ (p. 100). While users of the Doyle model needed to set the parameters for the community they were modelling, the ASSA models are explicitly aimed at modelling the South African epidemic, with later models disaggregating the outputs by province.

The starting point of the ASSA600 model was the 1985 South African population, known from a census conducted that year. The model was calibrated to reported AIDS cases in 1995 and antenatal HIV prevalence, derived from annual surveys by the Department of Health for 1994–1997. Dorrington described the calibration of the model as ‘perhaps inevitably a little more art than science’. The ASSA modellers aimed to produce a population estimate for 1996, national mortality rates for 1998, a projection of antenatal clinic HIV prevalence rates and a projection of national fertility rates.

Over the next decade, ASSA600 had several successors: ASSA2000, ASSA2002 and ASSA2008. From 2000, the suffix indicates the latest year of the empirical, primarily antenatal survey, data against which the models were calibrated (i.e. not the year they were published). The goal was to fit the known empirical data, estimate past unknown and project future, demographic and HIV outputs, such as population size, non-HIV and HIV mortality, HIV prevalence and incidence. From ASSA2002, the effects of antiretroviral treatment were incorporated.^5,6^

The ASSA models are widely cited. Besides being comprehensive, they have also been open and easily accessible. As Johnson explains, the ‘Excel interface of the publicly-available model is appealing to many non-modellers’.^18^

The latest ASSA model has been calibrated with data only as far as 2008. In recent years, the ASSA models too have been superseded, most notably by the THEMBISA model.^18^ This combines the features of three other models, besides the ASSA model. The model world complexity is substantial, including more realistic sexual behaviour ‘calibrated to marriage data and cross-sectional data on numbers of partners’, ‘more determinants of mother-to-child transmission’ and ‘most of the new strategies for preventing and treating paediatric HIV’. For example, it features CD4 count staging instead of clinical staging as in the ASSA models and allows for earlier antiretroviral initiation. It includes newer prevention interventions such as male medical circumcision, pre-exposure prophylaxis and ‘WHO options B and B+ for prevention of mother-to-child transmission’. In contrast to the ASSA models, it takes into account change in risk behaviour by people over time.

The Joint United Nations Programme on HIV and AIDS (UNAIDS) has also produced widely used models. In the 1990s, UNAIDS used Epimodel – developed in 1987 by the Global Programme on AIDS – for its global, regional and country HIV projections.^19^ This was eventually replaced by Spectrum, developed by the erstwhile Futures Group (now Avenir Health), and the Estimation and Projection Package (EPP), since combined into one programme.^20,21,22^

The model provides a user interface that takes a range of inputs, for example, base year population by age and sex, fertility rates, life expectancy (AIDS and non-AIDS), migration rates, number of people on antiretrovirals, number of people on cotrimoxazole and about a dozen or so more (see Table 1).^22^ It then aggregates all cases in the population aged 15 to 49 years and fits a non-age-structured population model to the historical aggregates, thereby inferring incidence and projecting outputs such as HIV infections and deaths.

Johnson^18^ writes:

The Spectrum/EPP model is used … in producing estimates of the global distribution of HIV, and therefore has the advantage of benefiting from a substantial body of international expertise in HIV epidemiology. However, the separation of the modelling of HIV incidence and demographic impact in this model does limit the ability of the model to make use of age-specific data in model calibration. [p. 6]

Spectrum/EPP is used to estimate official estimates for every country in the world every two years for the United Nations Population Division; it serves an important purpose, providing rough estimates of HIV prevalence and mortality where none would otherwise be available. The model is also used to analyse the long-term impact and cost of interventions, though as Johnson says, it is ‘limited in its ability to evaluate the impact of HIV prevention strategies and make long-term projections’. Where countries have developed high-quality specialised models, such as the THEMBISA model for South Africa, it makes more sense to use these.

## Modelling when to start treatment

In 2009, Granich et al. at the WHO presented two models.^14^ The first model is a population-level transmission model (implemented deterministically) that calculated the long-term dynamics of the HIV epidemic based on different treatment strategies. The second model (implemented stochastically) investigated the effect on R0 – ‘the number of secondary infections resulting from one primary infection in an otherwise susceptible population’ – of different treatment strategies applied to an hypothetical person.

The paper argued that, in South Africa, a policy of universal testing coupled with immediate treatment for adults found to be HIV-positive would effectively eliminate the epidemic. In particular, they estimated that HIV incidence could drop to less than 0.1% per year by 2016. They also costed the strategy.

The paper caused great excitement and controversy. It has been cited, according to Google Scholar, 1640 times (as of 11 March 2017). We know of no other HIV model that has been cited as often, which is extraordinary considering the simplicity of the models: there is no gender or age structure. Perhaps this simplicity, coupled with the strongly stated message the authors conveyed, engaged readers across multiple disciplines and accounted for much of the interest taken in the paper. The paper also encouraged a flurry of other models that looked at the same question.^23^

Even 4 years later, a detailed set of microsimulation models by Hontelez et al. was published, trying to answer the same question as Granich et al.^14^ The modellers developed ‘nine structurally different mathematical models of the South African HIV epidemic in a stepwise approach of increasing complexity and realism’.

The simplest resembled the Granich model. The most complex included ‘sexual networks and HIV stages with different degrees of infectiousness’. Hontelez et al.^24^ defined ‘universal test-and-treat’ as annual screening and immediate treatment for all HIV-positive adults, starting at 13% in January 2012 and scaling up to 90% coverage by January 2019. Elimination of the HIV epidemic was defined as incidence below 1 per 1000 person-years.

It is controversial whether addition of complexity to models improves them. For example, one of the authors of the Granich et al. paper, Brian Williams, has written:

Hontelez et al. suggest that the [then] current scale-up of ART at CD4 cell counts less than 350 [cells/mm^3^] will lead to elimination of HIV in 30 years. I disagree … and believe that their more complex models rely on unwarranted and unsubstantiated assumptions.^25^

The Granich model and the ensuing attempts by other modellers to verify, refute or improve upon it raise important questions about what we are trying to achieve with modelling. The original paper is the one that was widely debated. Even though it could be improved, it answered the question of whether a test-and-treat policy had the potential to massively reduce incidence. Most subsequent models agreed with that of Granich et al. that universal test-and-treat would substantially reduce new infections but not as quickly as they proposed. The assumption of rapid scale-up of treatment coverage and significant viral suppression in those failing treatment were, perhaps, too optimistic.

## Models targeting particular policy conundrums

Interventions other than antiretroviral treatments have also been modelled. There are numerous such models, and here we briefly note some without describing their model worlds.

The results of a randomised controlled trial that compared infection rates in circumcised versus uncircumcised men in Orange Farm^26^ were used to calculate that this intervention could prevent between 1.1 and 3.8 million infections as well as 0.1 to 0.5 million deaths over a 10-year period in sub-Saharan Africa.^27^ A comparison of the cost-effectiveness of treatment as prevention, treatment (solely for the benefit of the patient) and circumcision concluded that although treatment as prevention was cost-effective, it was less so than treatment or circumcision.^28,29^

Modelling the introduction of pre-exposure prophylaxis (PrEP), researchers found that it could avert 30% of new infections in ‘targeted age groups of women at highest risk of infection’. However, they also found that the cost-effectiveness of PrEP relative to treatment would decrease rapidly as treatment coverage increased.^30^ Another group had more optimistic results modelling PrEP in serodiscordant couples (although it is unclear how a model can address whether antiretrovirals should be given to the HIV-negative or HIV-positive partner in a relationship).^31^ They concluded:

Although the cost of PrEP is high, the cost per infection averted is significantly offset by future savings in lifelong treatment, especially among couples with multiple partners, low condom use, and a high risk of transmission. [p. 1]

Another model found that treatment plus PrEP was more effective than either strategy alone but would also produce high prevalence of drug resistance.^32^ Hallett et al. investigated the use of PrEP for seronegative partners in stable serodiscordant partnerships, as an alternative or adjunct to treatment for the HIV-positive partner.^31^

Sexual behaviour – such as condom use, number of partners, concurrency, and transactional sex – has been widely modelled.^33,34,35,36,37,38^ Models developed by the ASSA researchers, for example, estimated that HIV incidence in South Africa dropped during the period from 2000 to 2008 and that increased condom use was the ‘most significant factor explaining’ this decline.^39^ The role of concurrency has however been contentious, with conflicting findings.^33,34,40^

Experimental interventions such as microbicides^41^ and vaccines have also been considered,^42^ and so has the role of treating sexually transmitted infections.^43^ For further references, see Johnson.^18^

Currently, models such as THEMBISA and Spectrum are being used to track progress towards national and global objectives, such as the UNAIDS 90-90-90 targets (90% of people with HIV diagnosed, 90% of people diagnosed on treatment and 90% of people on treatment virally undetectable),^44^ as well as elimination of mother-to-child transmission.^45^

## Discussion

The distinction between model worlds and the technical implementation of models is useful for demystifying modelling and perhaps allows more people to participate in model construction and critique, and hence reach better informed decisions on the policy implications of models. While models, with their complex equations and computer code, might be impenetrable to all but specialists, the conceptual ingredients – the model world – should be accessible to a wide audience.

The earliest models of the South African HIV epidemic projected prevalence and mortality over time, a task that remains useful today. New models were subsequently developed to estimate the effects of interventions, for example, how antiretroviral treatment would reduce mortality (ASSA2002 interventions model) or how it would reduce new infections (the Granich model).

The challenge facing modellers was summarised by Dorrington^5^:

Estimating the exact impact of HIV/AIDS on mortality is not a simple task since there are many uncertainties surrounding the dynamics of the spread of the virus and subsequent passage to death. In addition there are difficulties in deciding on the level of overall mortality in South Africa since not all deaths are registered. However, determining an *order of magnitude of the impact* is well within the capabilities of a trained demographer. (our emphasis) (para. 7)

Models, even simple ones, can shed light on ‘big picture’ questions. They cannot be used to provide precise predictions of the long-term future. Models can also provide plausible estimates of unobserved epidemic indicators and assist with planning for the short-term future. These benefits and limitations of models should be kept in mind before deciding to add complexity to model worlds, and consequently model implementations.

Modelling is still an evolving component of biomedical science. Perhaps, as we argue in Appendix 1, a key factor in advancing consensus in how models are assessed, especially with societal implications, is a more inclusive interdisciplinary approach to defining and debating ‘model worlds’, and ‘model world scenarios’, the conceptual aspects of modelling that should be accessible to everyone with an interest in the HIV epidemic. This should lead to improved models that contribute more robustly to policy discussions.

## Appendix 1 A history of controversies involving human immunodeficiency virus (HIV) models in South Africa

### 1. Introduction

Disputes over models of the human immunodeficiency virus (HIV) epidemic have been public, vociferous, fraught and acrimonious. In this article, which supplements our article reviewing the main models of the South African HIV epidemic, we describe these debates. It is perhaps strange that the equations of obscure spreadsheets or highly technical computer code, understood only by a few specialists, should occupy so much attention in the media. But the stakes have been high, with millions of lives at risk. Also, as we explain in the main article, while, on the one hand, the technical workings of models might be understood by a few, on the other hand, the *model world*, that is, the conceptual realm of explicitly declared rules that match, in a simplified way, some aspect of the real world, can be understood by many.

### 2. The effect of models on policy in the 1990s

In 1990, although less than 1% of pregnant women attending public antenatal facilities in South Africa tested positive for HIV, modellers had predicted explosive growth during the subsequent decade, unless significant interventions were mounted. Indeed, by 2000, antenatal prevalence had increased to over 24%.^1^ This failure to control the epidemic might suggest that modelling had no useful influence on policy, but in fact the situation was more complex.

Concern about the growing HIV epidemic, based on modelling data and antenatal survey results, led to the formation of a body called the Networking HIV, acquired immune deficiency syndrome (AIDS) Community of South Africa (NACOSA) in 1991.^2^ In the transition to democracy in 1994, the African National Congress released a national health plan. It cites demographic modelling projections, stating that credible predictions ‘indicate that by the year 2005, between 18% and 24% of the adult population will be infected with HIV’ and that the ‘cumulative death toll will be 2.3 million, and that there will be about 1.5 million AIDS orphans’ (p. 30).^3^ But the plan’s recommendations, while cognisant of the rights of people with HIV, were mostly broad and vague, which, to be fair, was largely a reflection of the lack of effective interventions available at the time.

The small but growing AIDS activist movement was also aware of the model projections. The Treatment Action Campaign (TAC) was formed in late 1998. There are few early documents of the organisation’s work still available, but a letter to the Pharmaceutical Manufacturers Association on 22 September 1999 notes that the HIV epidemic is an ‘unprecedented health crisis in South Africa’ and ‘3.5 million people are already infected with HIV and it is estimated that 150,000 people die of AIDS related illnesses every year’.^4^ These figures were likely obtained from a model by the Actuarial Society of South Africa, such as ASSA600.^5^

### 3. The Medical Research Council report

A controversy with a model at its centre erupted in 2001. Dorrington et al. produced a technical report under the auspices of the South African Medical Research Council (MRC) that analysed mortality data collected by government agencies. The researchers also compared the mortality data with the projections of the ASSA600 model.^6^

The preface written by the president of the MRC at the time, Malegapuru Makgoba, stated there had been a shift in the age pattern of mortality in the country ‘from the old to the young over the last decade particularly for young women – this is a unique phenomenon in biology’, and ‘this shift in mortality fits several AIDS models’. Makgoba wrote that the ‘future burden’ of the epidemic was ‘broadly predictable from the models with reasonable confidence over the next decade’ (p. 4).

The study investigated trends in reported deaths until 1996 based on data from Statistics South Africa (Stats SA), compared with more recent data (mid-1997 to September 2000) from the population register of the Department of Home Affairs. These empirical data were then compared with the outputs of the ASSA600 demographic model ‘to assess the consistency of the empirical data with the model projections’ (pp. 8–9). The authors used standard techniques for adjusting the data to take into account under-reporting of deaths.

The data showed a ‘steady increase in adult mortality in the 1990s’. Women aged between 25 and 29 years had a 3.5 times higher death rate in 1999/2000 than in 1985 (p. 5).

The authors compared the empirical data with the projections of the ASSA600 demographic model, which they described as a ‘behavioural demographic component projection model, which models the heterosexual epidemic for the country as a whole, ignoring race and geographical heterogeneity’ (p. 19). The model was calibrated to reproduce the results of antenatal HIV surveys up to 1997.

The model projected antenatal infections between the values found for the 1999 and 2000 antenatal clinic surveys. It estimated that there would be between 4 and 7 million AIDS deaths from 2000 to 2010 in the absence of any interventions (behavioural change or treatment). The authors wrote that: ‘given the pattern of deaths exhibited by the ASSA600 model … the … estimate of non-AIDS deaths is probably a little on the low side … and the AIDS deaths a little exaggerated.’ (p. 24)

They also considered and critiqued the outputs of three other models: the Doyle one discussed in our main article,^7^ the one by the United Nations and the one by the US Bureau of Census (pp. 24–25).

The report noted limitations of both the available data and models. Its recommendations included proposals for improving both. It stated: ‘Considering these different sources of information, it seems highly probable that about 40% of the adult South African 1999/00 mortality in the 15–49 age group is due to HIV/AIDS.’ (p. 37).

It briefly considered interventions to mitigate the effect of the epidemic: AZT for mother-to-child transmission prevention, promoting increased use of condoms and a national campaign to treat sexually transmitted infections. The authors concluded that the interventions ‘can make a significant difference to the course of epidemic, although it will still exact a heavy toll’ (p. 38).

They also wrote: ‘Unfortunately the ASSA600 model was not designed to model the impact of antiretroviral therapies. Provided these drugs could be implemented successfully they could have a significant impact on the future prevalence levels.’ (p. 38)

(Note: the interaction between antiretrovirals, incidence, prevalence and mortality is complex and still not fully resolved by today’s models.)

The report was written against the background of the acrimonious debate in South Africa over the cause of AIDS, the size of the epidemic and whether antiretrovirals should be introduced in the public health system, both for mother-to-child transmission prevention as well as treatment (for a history of the AIDS denialist era, see Cameron).^8^ In particular, in March 2001, the Presidential AIDS Advisory Panel Report had been released.^9^

This panel, constituted by President Mbeki, consisted of a roughly equal number of AIDS denialists and conventional scientists. It was criticised for promoting AIDS denialism.^10^ The panel’s report contained a very short section on modelling (p.44), essentially noting a fundamental disagreement on their utility. It also stated in a section on epidemiology that repeated requests for ‘reliable data and statistics on the magnitude of the AIDS problem or even HIV prevalence’ (p. 45) had not been provided to the panel. Yet, the MRC report did provide this, as did many other reports available during the time of the panel’s deliberations, such as the Department of Health’s annual antenatal clinic studies.

The MRC report was carefully researched and showed the growing impact of the epidemic on adult deaths. However, the MRC board, together with the Minister of Health, stopped or delayed its publication, possibly ‘because it contradicted President Thabo Mbeki’s view that the epidemic was being vastly exaggerated and that there were other, larger causes of death’.^11^ However, findings from the report were leaked to the media. It was subsequently officially released in October 2001.^12^ The tensions surrounding the MRC study are illustrated by a news report: ‘[*The study*] prompted a whole new furore around AIDS statistics and the reliability of MRC research. Stats SA was then used by government to rubbish the MRC report, a move that was yesterday slammed by one of the authors of the MRC report, University of Cape Town Actuarial Science professor Rob Dorrington. Pointing out that he was speaking in his personal capacity, Dorrington said it was a great shame that Stats SA had decided to trash the report. “It is clear that they have a limited understanding of the estimation process and model. Their (Stats SA) presentation was riddled with half truths and misunderstandings”.’^9^

Instead of acting on the report’s concerning findings, the state’s response was to try to determine who leaked it. On 17 April 2002, Independent Newspapers published quotations from a letter obtained by reporter Lynne Altenroxel and written by the Minister of Health, Manto Tshabalala-Msimang, to the chair of the MRC board, Taole Mokoena, on 17 September 2001.^13^ Tshabalala-Msimang wrote, ‘this is not the first time that the MRC president has acted against government’.

She continued: ‘You will recall that when the president of South Africa established a website for the members of the Presidential Advisory Council on AIDS to debate their different points of view, the MRC president was instrumental in establishing a separate website for the orthodox scientists, under the umbrella of the MRC.’^13^

She further wrote: ‘The [*health department*] director-general [*Ayanda Ntsaluba*] advised the MRC president and his team not to release the report until the report had been presented to the minister of health and the cabinet.’^13^

Tshabalala-Msimang called for ‘corrective action’ to be taken. Makgoba was accused of being the source of the leak, but a subsequent investigation cleared him and three other MRC members. An earlier report by Independent Newspapers alleged that a private consultancy had been paid to find out the source of the leak.^13^ Makgoba soon resigned from the MRC and became the vice-chancellor of the University of KwaZulu-Natal.

Dorrington would later write that the only institution that seriously questioned the finding by the MRC study that AIDS was the largest cause of mortality in South Africa by 2000, responsible for 25% of all deaths, was Stats SA. But, wrote Dorrington, ‘[*Stats SA*] have not produced any statistics of their own and have not claimed that the figure should in fact be lower’.^14^

Stats SA officials attempted to discredit the Actuarial Society of South Africa (ASSA) model results. The institution released a press statement claiming that the model gave lower projected AIDS mortality by 2010 (1 to 2 million deaths vs. 5 or 6 million, in the absence of antiretroviral treatment) simply by changing the model’s assumptions. But as Dorrington pointed out, Stats SA failed to calibrate the model under their assumptions to known prevalence data.^14^

### 4. Mother-to-child transmission prevention court case

When the TAC launched with a small protest at St George’s Cathedral in Cape Town, one of the protesters’ demands was for the ministers of health and finance to meet with AIDS organisations to ‘plan for resources to introduce free AZT for pregnant mothers with HIV/AIDS’.^15^

Over the next 4 years, the TAC tried to convince the South African government to implement a countrywide mother-to-child HIV transmission prevention programme. The organisation, along with several others, proceeded with litigation against the national and provincial health ministers, eventually winning a seminal judgement at the Constitutional Court in July 2002. The court ordered the state to: ‘devise and implement within its available resources a comprehensive and co-ordinated programme to realise progressively the rights of pregnant women and their newborn children to have access to health services to combat mother-to-child transmission of HIV.’^16^

In this and subsequent TAC litigation, we find clear examples of modelling being used to make an argument for policy changes to increase access to antiretroviral medicines.

Evidence put before the court by the TAC included an affidavit by Nicoli Nattrass, an economist at the University of Cape Town. She concluded that the: ‘cost to the health sector of [*mother-to-child transmission prevention*] programmes … is less than the costs of treating all children born HIV+ in the absence of a … programme. This is true for all … of the … programmes discussed here.’^17^

Nattrass performed costing analyses in her affidavit, and cited similar work by other researchers. Her model was relatively simple compared with most of those discussed here, but Nattrass’s affidavit offered compelling arguments in favour of implementing mother-to-child transmission prevention. Although the role of Nattrass’s affidavit in the court’s decision is not mentioned explicitly in the court’s judgement, her submission made it practically impossible for the state to offer a coherent financial argument against implementing the programme (p. 31).^16^

### 5. Competition commission complaints

In 2002, the TAC lodged a complaint with the Competition Commission against two pharmaceutical companies, GlaxoSmithKline and Boehringer Ingelheim, over what the organisation called the excessive pricing of the antiretroviral medicines zidovudine (AZT), lamivudine and nevirapine.^18^

Here again an expert affidavit describing the results of several models was placed on record.^14^ The affidavit, written by Dorrington, explained the impact of HIV: ‘According to the models referred to above, well over five million people are currently infected with the virus and, unless they receive treatment that would increase their life expectancy, most of these people will die within the next 10 years. It is clear that HIV/AIDS is estimated by all demographers outside government to be having a devastating effect on the population and is undoubtedly the leading cause of death these days in South Africa.’ (p. 7)

In 2003, the TAC reached a settlement with the two companies that allowed generic manufacturers to sell the drugs in competition with them, not only in South Africa but also in sub-Saharan Africa.

The TAC followed up with other successful complaints and actions to lower antiretroviral prices. The effect on drug prices was profound: when Judge Cameron began taking antiretrovirals in 1997, the monthly cost of his regimen was R3419. By 2008, the standard regimen in the private sector cost under R240 per month.^11^ The Dorrington affidavit played a small but significant role in this.

### 6. Pushing for the state to treat

After the TAC won the mother-to-child transmission prevention court case, the organisation stepped up its demand for antiretrovirals to be made generally available in the public health system for the treatment of HIV.

To make the case for this much more expensive and vast programme, a TAC researcher, with the assistance of the ASSA model developers, Nattrass and others, calculated the cost of a countrywide treatment programme using the outputs of the ASSA2000 demographic model. The article concluded that implementing treatment would incur substantial direct costs but potentially provide long-term savings from reduced hospitalisations and treatment of opportunistic infections. The publication of this article in 2003 was at the height of the conflict between the TAC and government over antiretroviral treatment.^19^ The lead story in one of the country’s leading weekly newspapers at the time, *Mail & Guardian*, describing this work, was ‘Counting the cost of three million lives’.^20^ Its findings were debated in subsequent issues of the *Mail & Guardian*.^21^

This was not the first such costing model. In October 2002, Boulle et al. modelled eight scenarios of a limited antiretroviral rollout.^22^ This research did ‘not explicitly link their numbers on treatment to an external demographic model’, but they estimated the number of people needing treatment in their model would be about 10% of new HIV cases in an ASSA model.^23^

Another case of a model being used to advocate for treatment arose in mid-2003. The government had established the Joint Health & Treasury Technical Team, which used a model to estimate the cost of implementing an antiretroviral treatment programme in the public health system, and the number of deaths that would be averted by such a programme. The Director-General of Health, Dr Ayanda Ntsaluba, presented the team’s findings to the Health MinMec (the national and provincial ministers of health) on 9 May 2003.^24,25^ The presentation considered an antiretroviral treatment programme scaled up over three years in public hospitals. It showed three scenarios: treating 20%, 50% and 100% of AIDS cases. In the 50% scenario, 600 000 people would be on treatment by 2008 at a cost of about R10 billion (about $1.3 billion at the 2003 exchange rate). According to slides not shown at the meeting, this scenario would ‘defer’ 733 000 deaths until after 2010, assuming that treatment led to ‘4–5 additional years of relatively illness-free life’ (an extremely conservative assumption, on hindsight).

What model was used and how the results were calculated remains out of the public domain. The presentation was supposed to be a secret. However, based on a review of costing models by Boulle et al.,^23^ it appears that this prescient costing model was likely developed by Fareed Abdullah, an official at the time in the health department, in March 2003.

The TAC obtained the presentation and leaked it to the media in July 2003. Accusations and counter-accusations followed. At the time, following pressure from TAC, including a civil disobedience campaign, negotiations for a treatment plan were taking place, at the National Economic Development and Labour Council (NEDLAC), between the state, labour, business and civil society organisations. Advocate Rams Ramashia, the Director-General of the Department of Labour, accused the TAC of breaching ‘state security’ and ‘undermining and possibly de-railing the NEDLAC process’.^24^

The TAC responded: ‘On a matter of such fundamental importance to millions of people’s lives, the Constitutional right of access to information and the Constitutional duties that govern public administration are paramount. The notion that state security has been breached is ludicrous: in fact it is the personal security of millions affected and infected with HIV that is threatened by government procrastination.’^24^

By April 2004, the state began providing antiretroviral treatment to people with AIDS in the public health system. The programme stuttered in its first few years as Mbeki and Tshabalala-Msimang continued to undermine it, for example, by promoting untested remedies as alternatives. Eventually, the programme scaled up rapidly and is the largest of its kind in the world.

Since 2008, there has been considerable debate about the optimal clinical stage at which to start antiretroviral treatment. As described in our main article, models, such as the one by Granich et al., have played a central role in this debate.^26^ Following the results of a clinical trial in 2015, the World Health Organization has recommended, and the South African government has adopted, a policy of universal access to treatment for all people with HIV.

### 7. AIDS denialists attack models

South Africa’s period of state-supported scepticism of the link between HIV and AIDS under President Thabo Mbeki lasted from the late 1990s until Mbeki was removed from power by his own party in 2008. It’s important to note that the governing party was not unified in its dismissal of the epidemic, and Mbeki’s position ultimately delayed rather than entirely prevented the implementation of the public sector antiretroviral treatment programme, which began in 2004. Throughout this period, there was a constant battle between the supporters of the scientific position and the AIDS denialists, with the former slowly becoming ascendant until AIDS denialism ceased to be a relevant political force in South Africa.^11^

Mathematical models were at the centre of this conflict. The AIDS denialist attack on modelling did not come from a scientist but from a journalist, Rian Malan. Well-known for his best-selling non-fiction book *My Traitor’s Heart*,^27^ Malan wrote an article in *Rolling Stone* in 2001, disputing that there was a large HIV epidemic in Africa including South Africa. The article questioned HIV testing methodology on the continent and essentially accused UNAIDS of cynically exaggerating the size of the epidemic.^28^ He followed this with articles in the British magazine *The Spectator*^29^ and the South African magazine *Noseweek*.^30^ A large part of the latter two articles was aimed at mathematical modelling of the epidemic, particularly the ASSA models.

A TAC researcher published a detailed rebuttal of Malan.^31^ Interestingly, no rebuttal approaching the detail of the TAC’s response was written by scientists with recognised expertise in demography, although Leigh Johnson, one of the main producers of the ASSA and subsequent models, assisted the TAC’s researcher. It appears that academics found Malan’s arguments so absurd that they were not worth more than a cursory occasional response in newspaper articles. This despite the fact that his three articles, which appeared in large-circulation popular publications, almost certainly were more widely read than the peer-reviewed publications on AIDS demographics in sub-Saharan Africa.

Malan’s articles had numerous errors. In *Noseweek*, he miscalculated the number of South African HIV deaths from a Stats SA report, ignoring that many deaths owing to AIDS were not officially classified as AIDS deaths. He therefore reached the incorrect conclusion that they were a small fraction of the ASSA estimates.

The Stats SA report in fact made it clear that if physicians wrote the cause of death as, for example, tuberculosis, then this was not classified as an AIDS death, even though many such deaths are AIDS-related. The report stated that extricating the HIV-related deaths from the other death categories is where ‘official statistics stop and research begins’ (p. 28).^32^ The TAC added: ‘Malan has not bothered with such research, which would be a very complex undertaking’.^31^

Mbeki mentioned Malan favourably in his 2004 State of the Nation speech, which a TAC researcher characterised thus: ‘It was not explicitly about HIV, but to anyone following the debate at the time, it was clear that Mbeki was grateful for Malan’s support on AIDS’.^33^

Running battles in print between Malan, activists and, to a lesser extent, scientists continued through the 2000s. In 2007, Malan published again in Noseweek, suggesting that the rise in recorded deaths was primarily owing to improved registration.^34^ Grebe^35^ pointed out on a website dedicated to refuting AIDS denialism, https://www.aidstruth.org/, that Malan continued to ignore the age pattern of deaths in South Africa, in which most recorded deaths were among young adults, as well as ‘the increase in the recorded deaths resulting from causes typically associated with AIDS’.

In the post-Mbeki, and thus post-denialist, period, Minister of Health Aaron Motsoaledi delivered a presentation in which one of his slides had erroneously substantially overstated the number of 2008 deaths. Malan pounced on this in the online news site Politicsweb. Besides correcting the error, Malan wrote, ‘[T]here is no apocalypse. No massive AIDS -related death surge. If anything, death registrations are stable’.^36^ Malan’s point was petty: Motsoaledi’s slide had mistakenly transposed two digits, reporting 756 062 instead of 576 062 AIDS deaths.

The argument continued when Malan published his book, *Resident Alien*, in 2009.^37^ It contained a chapter that reaffirmed his position, disputing there was a large HIV epidemic. The Daily Maverick, a popular South African news site, gave it a favourable review, and then published a critical reply by the TAC.^38,39^

After the removal of Mbeki from office, AIDS denialism no longer had any political force. Public debates over the size of the HIV epidemic receded.

### 8. The impact of AIDS denialism

After the rollout of antiretroviral treatment and the termination of Thabo Mbeki’s presidency, two studies were conducted that calculated the loss of life owing to AIDS denialist policies.

Nattrass^40^ used the ASSA2003 demographic model to estimate that if the national government had used antiretrovirals for mother-to-child transmission prevention and treatment of people with HIV at the same rate as the Western Cape province ‘which defied national policy on ARVs’, then 171 000 HIV infections and 343 000 deaths could have been prevented, just between 1999 and 2007.

A few months later, Chigwedere and colleagues at Harvard University used a different methodology but reached similar conclusions.^41^ They considered what the South African government could have achieved had it scaled up treatment coverage from 5% in 2000 to 50% in 2005 instead of 3% to 23%. These estimates were actually more modest than what was achieved by Botswana or Namibia. Using a UNAIDS model accounting for the period 2000 to 2005, they concluded that delayed treatment caused 2.2 million lost person-years and over 330 000 deaths, and delayed mother-to-child transmission prevention caused over 35 000 excess infections and 1.6 million lost person-years. Both studies have been cited in an argument that Mbeki and the late South African Health Minister Manto Tshabalala-Msimang should have been prosecuted.^42^

### 9. Conclusion

This article has shown that mathematical models have been at the centre of policy debates and decision-making in the context of the South African HIV epidemic. In the early 1990s, models acted as a warning sign of the pending mortality that would be caused by the disease. In the late 1990s until the mid-2000s, modelling was a key point of discussion in the AIDS denialist controversy that characterised the government’s response to the epidemic. Modelling also informed discussions on the relative efficacy of treatment and prevention options.

But, these examples also show the limitations of the influence of modelling over public policy. Despite the warnings of the early 1990s, little was done to stem the rise of HIV infections. And in the 2000s, the models were simply disputed by the AIDS denialists, so that antiretroviral treatment was delayed until 2004, and then only after an immense conflict between the state and AIDS activists, of which the dispute over modelling results was but one aspect. Also, no AIDS denialists have been held accountable for their role in hundreds of thousands of avoidable deaths, despite the estimates of Nattrass and Chigwedere et al.

Even the Granich model, cited an order of magnitude more often than any other model, had a limited effect on public policy. It was not until the publication of the HPTN 052 trial that there was consensus that test-and-treat would be effective at reducing new infections,^43^ and it was not until the results of the START randomised control clinical trial in 2015 that there was consensus that antiretrovirals should be provided to all with HIV irrespective of CD4 count.^44^ Although models have informed these debates, they do not carry the same weight as other forms of evidence in medicine, especially randomised controlled trials.

Perhaps, if consensus is reached on which modelling techniques produce the most robust estimates of past outputs and future projections – in other words if the science of modelling improves greatly – future models will be more effective at changing policy. We are aware of no developments that suggest this is likely to happen. Nevertheless, as a means of exploring future scenarios and understanding the epidemic better, models have played a vital role.

### 10. Acknowledgements

#### Competing interests

Geffen was with the Treatment Action Campaign from 2000 to 2013 and an active participant in some of the debates discussed in this article.

#### Authors’ contributions

N.G. conceived and drafted the article. N.G. and A.W. reviewed and edited the article.

### 11. Summary of Appendix 1

Mathematical models have helped describe and project the South African human immunodeficiency virus (HIV) epidemic. They have also informed, and been the subject of, public debates. We describe the main policy debates in which models had a crucial role, explaining how they were used to inform these debates, and we discuss the limits of how they influence policy. In the early 1990s, models were used to warn of the impending epidemic. The models of the early 2000s informed debates on treatment for people with acquired immune deficiency syndrome (AIDS) and prevention of mother-to-child transmission. Models were also at the centre of the AIDS denialist controversy. In more recent years, models have played a key role in the debate on when to start treatment.
